# Angular correction methodology and characterization of a high‐resolution CMOS array for patient specific quality assurance on a robotic arm linac

**DOI:** 10.1002/acm2.14110

**Published:** 2023-08-02

**Authors:** Muhammad Ramish Ashraf, Jochen Krimmer, Laszlo Zalavri, Xuejun Gu, Lei Wang, Cynthia Fu‐Yu Chuang

**Affiliations:** ^1^ Department of Radiation Oncology Stanford University Palo Alto California USA; ^2^ IBA Dosimetry GmbH Schwarzenbruck Germany

**Keywords:** cyberknife, detector array, high‐resolution, patient specific QA

## Abstract

**Purpose:**

To develop an angular correction methodology and characterize a high‐resolution complementary metal‐oxide‐semiconductor (CMOS) array for patient specific quality assurance on a robotic arm linear accelerator.

**Methods:**

Beam path files from the treatment planning software (TPS) were used to calculate the angle of radiation beam with respect to the detector plane. Beams from multiple discrete angles were delivered to the CMOS detector array and an angular dependency look up table (LUT) was created. The LUT was then used to correct for the angular dependency of the detector. An iso‐centric 5 mm fixed cone, non iso‐centric multi‐target fixed cone, 10 mm Iris and a multi‐leaf collimator (MLC) based collimated plan were delivered to the phantom and compared to the TPS with and without angular correction applied. Additionally, the CMOS array was compared to gafchromic film and a diode array.

**Results:**

Large errors of up to 30% were observed for oblique angles. When angular correction was applied, the gamma passing rate increased from 99.2% to 100% (average gamma value decreased from 0.29 to 0.14) for the 5‐mm iso‐centric cone plan. Similarly, the passing rate increased from 84.0% to 100% for the Iris plan and from 49.98% to 98.4% for the MLC plan when angular correction was applied. For the multi‐target plan, applying angular correction improved the gamma passing rate from 94% to 99.6%. The 5 mm iso‐centric fixed cone plan was also delivered to film, and the gamma passing rate was 91.3% when using gafchromic film as the reference dataset, whereas the diode array provided insufficient sampling for this plan.

**Conclusion:**

A methodology of calculating the beam angle based on the beam path files was developed and validated. The array was demonstrated to be superior to other quality assurance tools because of its sub‐millimeter spatial resolution and immediate read out of the results.

## 1 INTRODUCTION

CyberKnife (Accuray, Sunnyvale, CA) is a radiosurgery system that uses a X‐band linear accelerator mounted on a robotic arm to deliver high doses of radiation with steep dose gradients.^1^ Due to the small fields (5 mm) and steep dose gradients, the need for accurate patient specific quality assurance (QA) is paramount and the requirements are more stringent as compared to conventional forms of radiotherapy.^2^ A detector array, which has minimal inter‐detector spacing, and small individual detector size is desired for patient specific QA. Current techniques commonly used to perform patient specific QA include gafchromic film,^3^ diode,^4^ ionization chamber array,^5^ and point verification using ionization chambers.^6^ Gafchromic films provide the highest resolution of any detector but require meticulous calibration and read‐out procedures, and the uncertainty associated with film is typically on the order 2%−3%, even if strict calibration and read‐out protocols are followed.^7^ In addition, a period of 24 h is usually required before readings can be obtained after irradiation. Commercially available diode and ionization chamber arrays typically do not provide sufficient spatial resolution to accurately QA treatment plans with field sizes <10 mm. At our institution, there is a significant number of treatments that employ small field sizes, therefore our ability to QA these plans with fast turn‐around time is limited. The aim of this study was to characterize a high resolution (0.4 mm pixel resolution) complementary metal oxide semiconductor (CMOS) detector array for patient specific QA on Cyberknife. The CMOS array (myQA SRS), manufactured by IBA Dosimetry (Schwarzenbruck, Germany), is commercially available for C‐arm linacs, but is undergoing extensive testing for use on Cyberknife. This is because the output of the array is highly dependent on the incidence angle of the beam with respect to the plane of the detector. This angular dependency can be accounted for on a C‐arm linac by using a gantry angle sensor mounted on the gantry. However, accounting for this angular dependency on a robotic arm linac is non‐trivial as most plans are not iso‐centric, and the 6‐degree robotic‐arm motion precludes the use of the angle sensor. Therefore, we present a methodology to account for the angular dependency based on the angles calculated from beam path files obtained from the TPS and characterize the CMOS array for patient specific QA on Cyberknife. Fixed cone, Iris and multileaf collimator‐based plans were measured with the CMOS array. The effect of the angular dependency was assessed by comparing gamma passing rates with and without the angular correction. A comparison was performed against the TPS, a diode array that is, currently used at our institution, and gafchromic film. Recently, Padelli et al.^8^ characterized the same CMOS detector array in terms of dose linearity, dose‐rate dependence, and angular dependency. However, the angular dependency of the array was not corrected for during patient specific QA. The authors measured multiple treatment plans but had to resort to ‘beam pruning’ that is, removing beams that were above a certain angle. To the best of our knowledge, this is the first instance of the CMOS detector being used for patient specific QA with the angular dependence accounted for on Cyberknife. Padelli et al. also noted the absence of radiological markers for fiducial tracking. The fiducial plate is necessary for accurate image‐guided radiation therapy (IGRT) set up on the treatment couch for the Cyberknife. However, given similar feedback from our institution, the detector now also includes a fiducial plate.

## 2 METHODS AND MATERIALS

### 2.1 CMOS detector array

**FIGURE 1:**
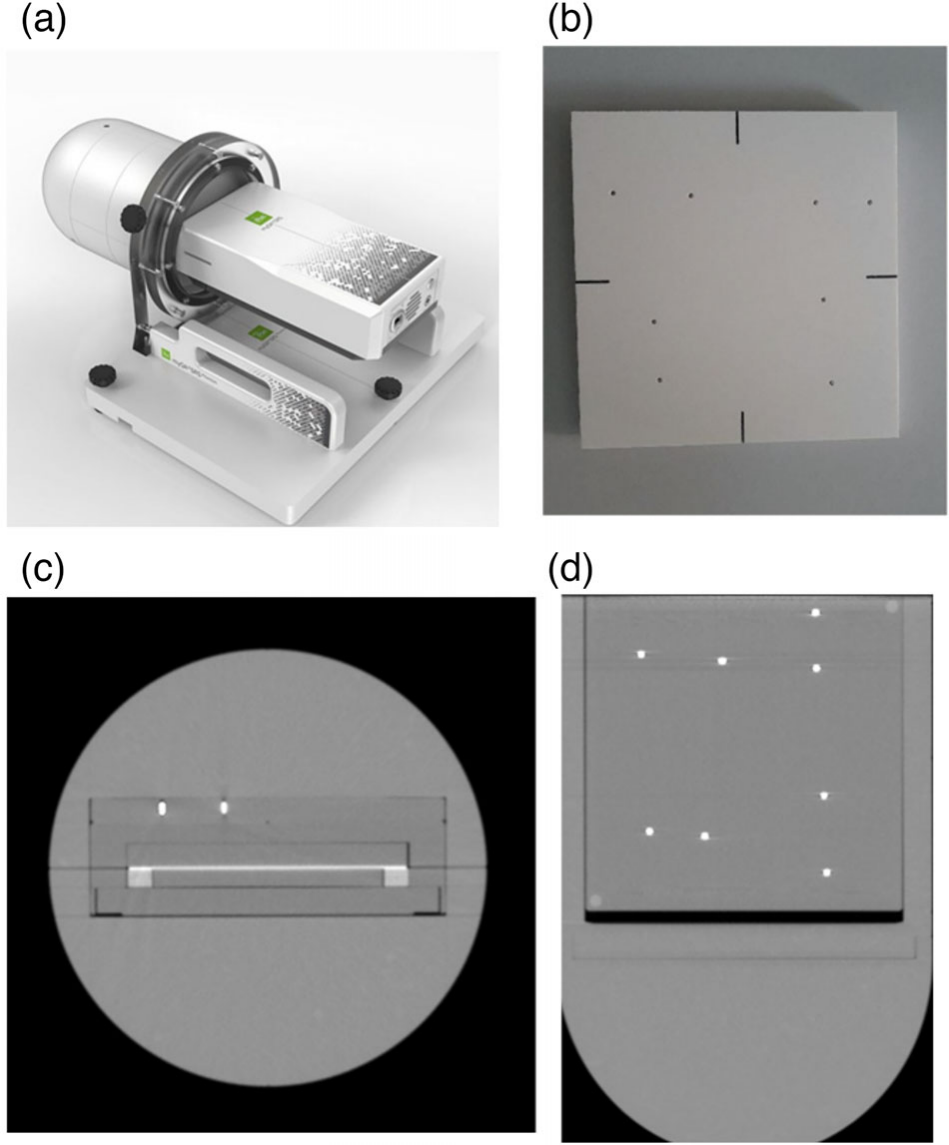
(a) shows a picture of the detector array inside the cylindrical housing. (b) shows an image of the fiducial plate. (c) and (d) show the front and top view of the detector along with the fiducial markers, respectively.

The myQA SRS is a solid‐state CMOS detector array with a pixel size of 0.4 mm × 0.4 mm and a total of 105 000 pixels. The pixels are contiguous, that is, the inter‐detector spacing is zero. This ensures that no interpolation is needed, and that the spatial resolution is comparable with film. The maximum field size that can be measured with the detector is 12 cm × 14 cm, which is sufficient for Cyberknife based patient specific QA, including MLC‐based plans. The detector array is housed inside a cylindrical Acrylonitrile Butadiene Styrene (ABS) enclosure. A fiducial plate can be affixed to the top of the detector. The fiducial plate consists of 8 fiducials which are placed according to Accuray's guidelines for accurate IGRT setup. The housing also has dedicated inserts for film and ion chambers. Figure 1 shows the myQA SRS along with the cylindrical housing and the fiducial plate. CT scan of the phantom with the fiducial plate affixed on top of the detector is also shown in Figure 1. The phantom was CT scanned with 120 kVp tube voltage and 350 mA tube current and metal artefact reduction turned on to account for streaking artefacts due to the fiducials. The voxel size was 0.65 mm^3^× 0.65 mm^3^ × 0.6 mm^3^.

### 2.2 Absolute dose calibration

The absolute dose was calibrated by irradiating the detector array with a 35 mm fixed cone beam at 0° (i.e., AP beam). The planned dose was exported in Dicom format from the TPS and compared against the measured output of the CMOS array. A small ROI enclosing the central detectors was used to establish the counts/cGy factor. The 35 mm cone provided sufficient flatness in the ROI. This factor was subsequently applied to all irradiation performed in this study. The pixel sensitivity map is provided by the vendor which enables a single calibration factor to be applied to all pixels in a 2D image.

### 2.3 Accounting for angular dependency

To assess the angular dependency of the detector array, a 35 mm cone was used at 0° (AP direction), and 500 Monitor Units (MU) were delivered to the array for each irradiation. The beam angle was kept constant, and the detector array was rotated inside the cylindrical housing from 0° to 130° for both clockwise and counterclockwise directions. No angles between 130° and 260° were measured because Cyberknife does not have any posterior beams due to the limitations of the robotic arm. Detector output at each angle was normalized to the detector response at 0°. The LUT was constructed by extracting the average response in the central area of the sensor for each beam angle relative to 0°. Currently, the same correction factor is applied to all pixels, independent of their location on the sensor area. A previous characterization of the sensor at a C‐arm Linac concluded that this type of angular correction is sufficient.^9^ Note that iso‐centric setup was used for this experiment because the angle between the radiation beam and the detector plan can be accurately known. The LUT would still be applicable to non‐isocentric plans.

### 2.4 Determination of cyberknife beam angles

Cyberknife treatment plans consist of beam paths which comprise of discrete positions in space called nodes. Beam path *xml* files can be obtained from the TPS which includes information about the discrete location of each node (i.e., beam source) and the order in which the nodes are traversed during irradiation. Furthermore, each node contains the description of one or more beams. The parameters for each beam are expressed in terms of *x*, *y*, *z* coordinates and *a*, *b*, *c* angles which correspond to yaw, pitch, and roll. In a pure isocentric scenario, the information from the *x*, *y*, *z* coordinates would be sufficient to calculate the incident angles on the sensor plane. However, in general the beams do not traverse the center of the sensor. Especially in cases with multiple targets, some beams would not intersect the sensor plane at all, and just stray radiation could be recorded in the detector. To cover all these cases, the directional information from the beams is used for the 3‐D rotation matrix *R*:

$$\begin{array}{ccc} R & = & R_{z}\left(\alpha \right)R_{y}\left(\beta \right)R_{x}\left(\gamma \right)=\left[\begin{array}{ccc} \cos\alpha & -\sin\alpha & 0\\ \sin\alpha & \cos\alpha & 0\\ 0 & 0 & 1 \end{array}\right]\\ & & \times \;\left[\begin{array}{ccc} \cos\beta & 0 & \sin\beta \\ 0 & 1 & 0\\ -\sin\beta & 0 & \cos\beta \end{array}\right]\left[\begin{array}{ccc} 1 & 0 & 0\\ 0 & \cos\gamma & -\sin\gamma \\ 0 & \sin\gamma & \cos\gamma \end{array}\right] \end{array}$$

where α, β, and γ correspond to angles *a, b*, and *c* (yaw, pitch, and roll). The beam direction vector (***u)*** can be computed using vector matrix multiplication of *R* and the unit vector, as shown below:

$$u=R\left(\begin{array}{c} 0\\ 0\\ -1 \end{array}\right)$$

The beam angle (***δ*)** can then be calculated using the formula below, where ***n*** is the surface normal to the detector plane.

$$\mathrm{Cos}\left(\delta \right)=\frac{u\cdot n}{\left|u\right|\left|n\right|}$$

This method of calculating the beam angle was validated by constructing an iso‐centric plan in the TPS and comparing the angle calculated using *a*, *b*, and *c* angular information to the beam angle calculated by using the *x*, *y*, and *z* coordinates.

In certain cases, especially for plans with multiple targets, the plane of the detector may need to be rotated such that most of the high dose regions are sampled by the detector. Therefore, if the phantom is rotated by an angle ω with respect to the *x*‐axis, the new normal vector would be:

$$n^{\prime }=\left(\begin{array}{ccc} 1 & 0 & 0\\ 0 & \cos\left(\omega \right) & -\sin\left(\omega \right)\\ 0 & \sin\left(\omega \right) & \cos\left(\omega \right) \end{array}\right)\left(\begin{array}{c} 0\\ 0\\ -1 \end{array}\right)$$

The beam angle ***δ*** would then need to be calculated relative to the new normal vector.

### 2.5 Patient specific QA: Assessing effect of angular correction

A 5 mm fixed cone, Iris (a variable aperture collimator consisting of two tungsten‐copper alloy banks rotated 30° apart),^10^ MLC plan and a fixed cone multiple target plan were measured in movie mode with a frame rate of 2 Hz. As mentioned above, the beam path xml files contain angular information for each delivered beam. Furthermore, the traversal order of beams in the file corresponds to the actual delivery in step‐and‐shoot mode. The signal in each acquired frame is analyzed to determine whether it is above a given threshold to identify the individual beam‐on periods. The number of registered beam‐on phases needs to be identical to the number of beams from the path xml file, so that the correct angular information is used. For each beam, the incident angle on the sensor plane can then be calculated as described above. For this angle, the corresponding correction factor (inverse of the angular dependent response in Figure 3a from the LUT is applied by performing scalar multiplication of the correction factor with the image matrix.

All plans were compared with the treatment planning software distribution using 2%/2 mm gamma criteria and a 10% low dose threshold. Comparison was performed with and without applying angular correction. For the multi‐met plan, a 30° in‐phantom rotation was used. The 5 mm cone plan was acquired with a diode array and gafchromic film as well. The purpose of this test was to demonstrate that the resolution of the CMOS array is more useful for small field patient specific QA than what is currently available in our clinic besides film. The diode array used in this study had a pixel spacing of 2.47 mm with individual detector size of 0.48 mm × 0.48 mm. The gafchromic film was irradiated using the film insert which can be inserted in place of the CMOS array in the cylindrical housing. Both modalities were compared with the CMOS array. The details of the treatment plans used in this study are shown in Table 1. It is worth comparing the effect of the angular correction on individual frames as well, as errors can be averaged out when cumulative comparison is performed. This analysis was performed by analyzing a subset of beams from the 5 mm fixed cone iso‐centric plan. The dose obtained from the reference point in the TPS was compared against the corrected and uncorrected dose at the same point in the measurement dataset for each individual beam.

**TABLE 1:** Monitor Units, dose and the number of beams used in each of the plans measured in this study.

Collimator	MU	Dose (Gy)	Number of beams
Fixed Cone (5 mm)	7368.00	20.05	67
Iris (15, 20 mm)	4855.40	14.15	95
InCise 2 MLC	14856.11	23.30	90
Fixed Cone (Multi‐Target)	23194.80	26.70	106

Abbreviation: MU, Monitor Units.

## 3 RESULTS

### 3.1 Absolute calibration and angular correction LUT

**FIGURE 2:**
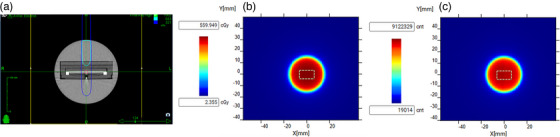
(a) shows the treatment plan that was used for absolute dose calibration. (b) shows the image obtained from the TPS dose distribution. (c) shows the image recorded by the CMOS array. White dotted lines represent the ROI that were used to obtain the counts/cGy factor. CMOS, complementary metal‐oxide‐semiconductor; ROI, region of interests; TPS, treatment planning software.

**FIGURE 3:**
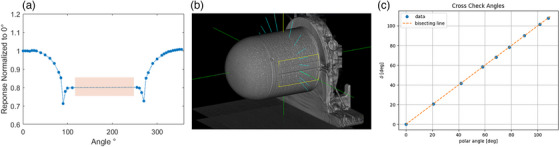
(a) Shows the response of the detector at different beam incidence angles normalized to the response at 0°. The measured angle range was between 0° to 130° and 230° to 360°. The shaded area indicates the region where no angular dependecy data was acquired. (b) shows an image of the plan used to validate the script which is used to calculate the beam angle at each node. (c) Shows a comparison of the angle δ calculated from the *a*, *b*, *c* angles versus the polar angles calculated from the *x*, *y*, *z* coordinates. In the present isocentric case the data are on the bisecting line indicating the 1 to 1 correspondence between both angles.

The treatment plan, TPS distribution and the CMOS image used to generate the absolute dose calibration is shown in Figure 2a, 2b and 2c, respectively. The counts/cGy factor was measured to be ∼ 15617 counts/cGy. The response of the detector with respect to different beam angles is shown in Figure 3a. It is worth noting that the angular response of the detector is symmetric around the IEC‐*z* axis. Errors up to 30 % can be observed for oblique angle, and the maximum errors occur at 90 ° or 270 °. Only data between 0°−130° and 230°−360° was acquired, as this covers most of the nodes that the robotic arm can deliver beam at. This measurement formed the basis for the LUT used for angular correction. The script used to parse the beam path files to determine the beam angle was validated by creating an isocenteric plan in the TPS and comparing beam angle (δ) calculated by using the *a*, *b*, *c* coordinates to the polar angle calculated by using *x, y, z* coordinates. Good agreement between the two provides assurance regarding the validity and reliability of the script. The plan is displayed in Figure 3b and Figure 3c shows the angle (δ) as calculated using the *a, b, and c* coordinates versus polar angle as calculated using the *x*, *y*, and *z* coordinates. Excellent agreement is seen between the two calculated angles.

### 3.2 Patient specific QA with and without angular correction

**FIGURE 4:**
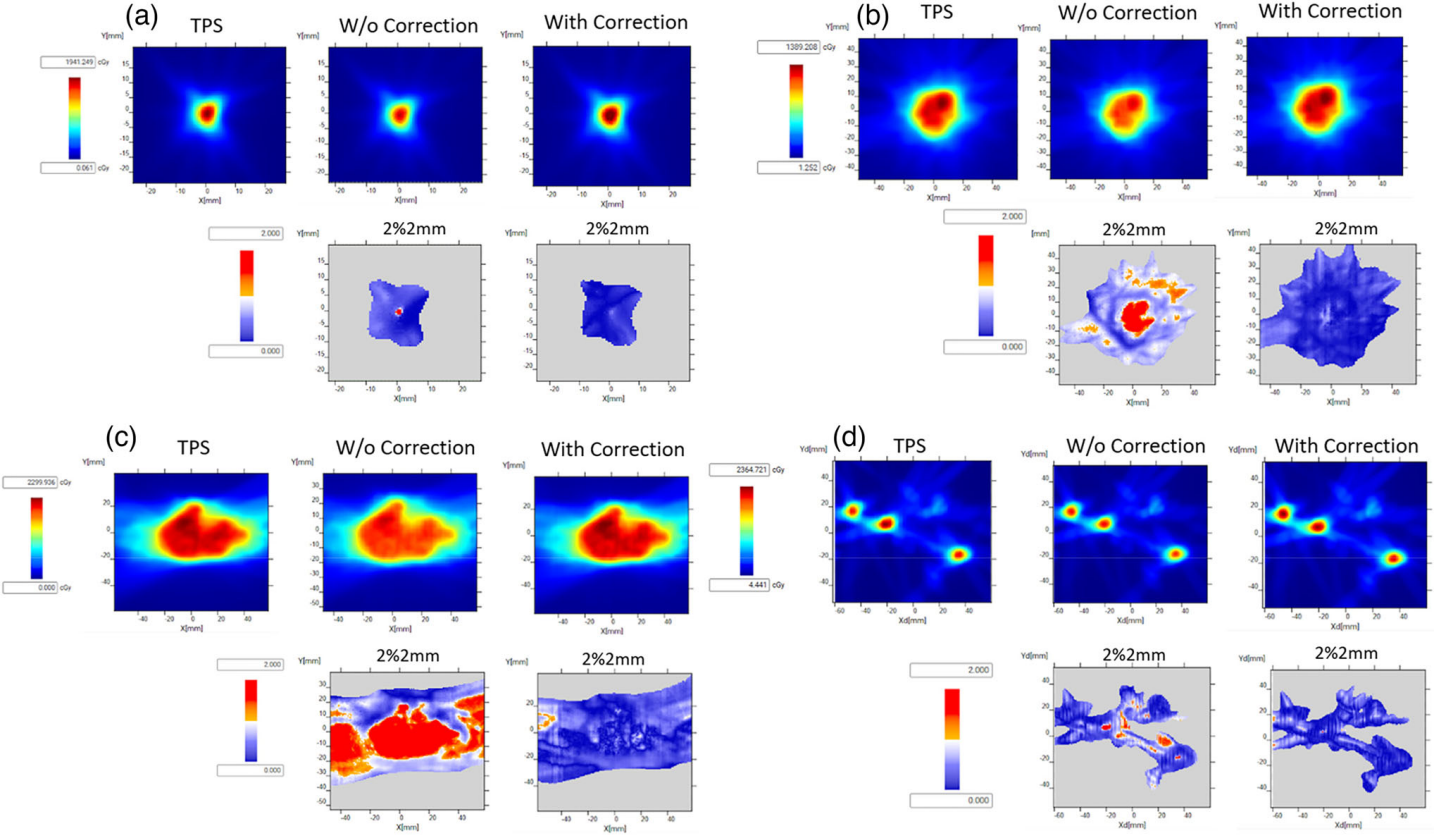
(a) shows a slice (corresponding to the physical depth of the sensor) out of the TPS generated dose distribution for the 5 mm fixed cone plan and the image acquired with the CMOS array with and without angular correction applied, respectively. Gamma maps are also shown with gamma criteria 2%/2 mm (10% dose threshold, global normalization). Without correction, the gamma passing rate was 99.2% (average gamma value = 0.29), and with correction the passing rate 100% (average gamma value = 0.14). (b)–(d) show the same dataset but for the Iris, MLC and the multi‐target plan. For the Iris plan, without correction, the gamma passing rate was 84.0 % (average gamma value = 0.83), and with correction was 100% (average gamma value = 0.23). For the MLC plan without correction, the gamma passing rate was 49.8 % (average gamma value = 1.22), and with correction the passing rate was 98.4 % (average gamma value = 0.31). For the multi‐met plan, the gamma passing rate increased from 94.0% to 99.6% when angular correction was applied. CMOS, complementary metal‐oxide‐semiconductor; TPS, treatment planning software.

Figure 4 shows the 2D gamma analysis of all the plans imaged in this study. Figure 4a show the 5 mm cone plan and the gamma comparison (2%/2 mm with 10% low dose threshold) with and without applying angular correction. Without angular correction, the gamma passing rate was 99.2% with an average gamma value of 0.29. When angular correction was applied, the gamma passing rate increased to 100%, with an average gamma value of 0.14. This small difference in gamma passing rate with and without the angular correction can be attributed to the small target size and iso‐centric nature of the plan. The small difference in the passing rate may also be a consequence of the gamma comparison metric which can sometimes mask dose difference errors.^11^ Nonetheless, the average gamma value decreased by 51%, when angular correction was applied. The effect of angular correction on individuals frame is presented in Table S1 in the supplementary data. The average difference from the reference dose for the corrected distribution was 2.74% (differences ranging from 9.91% to 0.76%) and for the uncorrected distribution, the average difference was −13.8% (differences ranging from −25.85% to −2.53%). Applying angular correction substantially improves the absolute dose agreement for individual beams. Note that some of these differences may be exaggerated due to the small field size and that the reference point for certain beams was in the steep dose gradient region.

Figures 4b and 4c show the gamma analysis for the Iris and the MLC plan, respectively. For both plans, the need for applying angular correction is apparent as the gamma passing rate improves significantly after the correction is applied. For the Iris plan, the gamma passing rate increased from 84% (average gamma value = 0.83) to 100 % (average gamma = 0.23). For the MLC plan, the gamma passing rate increased from 49.8% (average gamma value = 1.22) to 98.4% (average gamma value = 0.31). The reason for the substantial increase in the gamma passing rate for MLC plan was due to the large size of the target. Figure 4d shows the gamma analysis results for the multi‐target plan with an in‐phantom rotation of 30°. The gamma passing rate increased to 99.6% (average gamma value = 0.25) from 94.0% (average gamma value = 0.45) when the angular correction was applied. This is a modest increase in the passing rate, but this can be attributed to the fact that the plan consisted of small, isolated targets yielding a result similar to the fixed 5 mm cone. For this plan, the plane of the detector was also rotated to 30°, which minimized oblique angles.

### 3.3 Comparison with a diode array and gafchromic film

**FIGURE 5:**
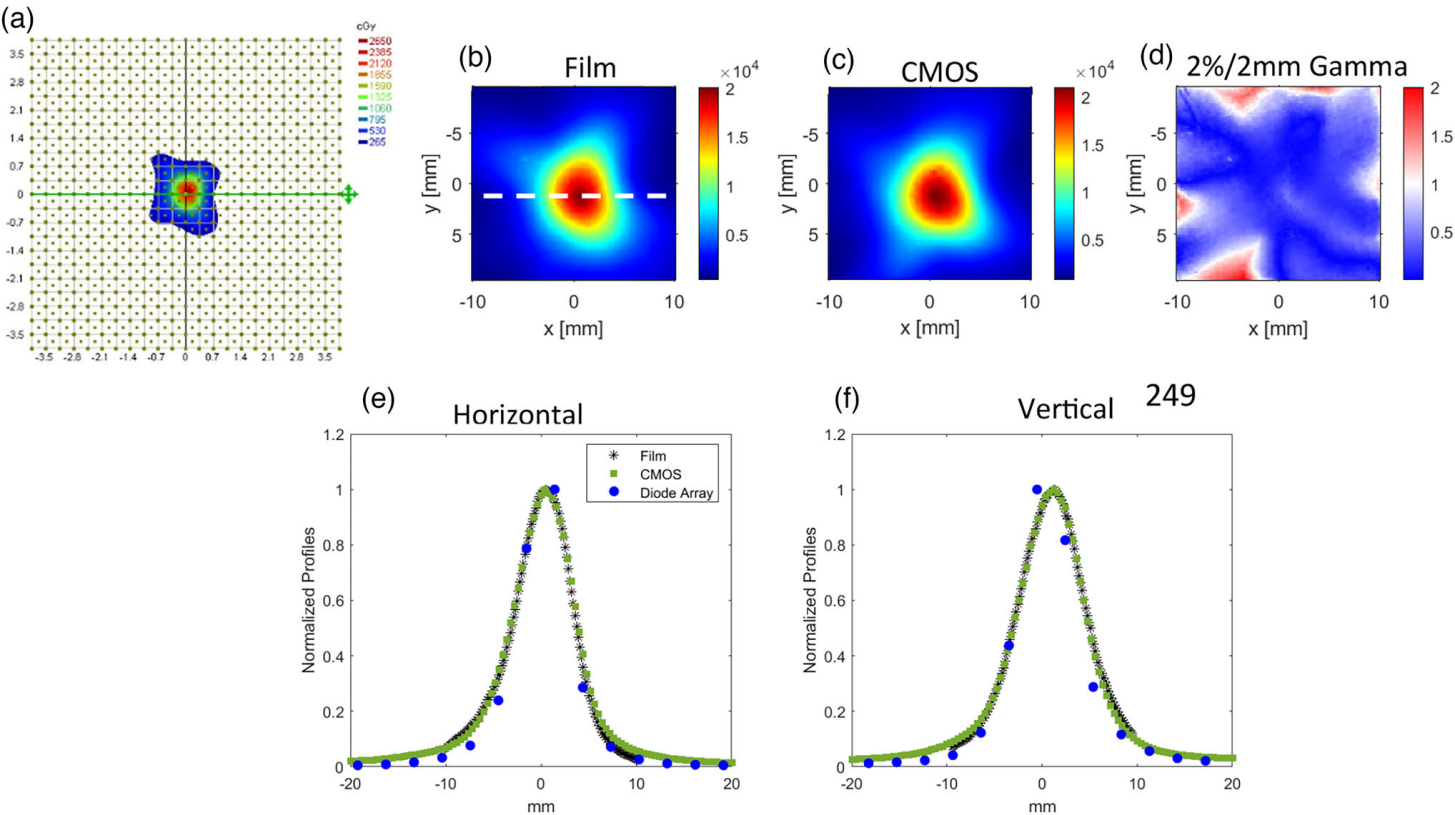
(a) shows the 5 mm cone plan acquired using the diode array. (b)–(c) show the film and CMOS dose distribution for the 5 mm plan. (d) shows the 2%/2 mm gamma comparison. The gamma passing rate was 91.3%, with film as the reference. (e)–(f) show the horizontal and vertical line profiles corresponding to the dotted white lines in the (b). Film and the diode array profiles are also shown for comparison. CMOS, complementary metal‐oxide‐semiconductor.

The 5 mm plan as measured by the diode array is shown in Figure 5a. The comparison of film and the CMOS array is shown in Figure 5b–d. Vertical and horizontal line profiles are shown in Figures 5e,f. The gamma (2%/2 mm) passing rate was 91.3% when comparing the CMOS array versus film as the reference dataset. The superior resolution of the CMOS array is evident in the line profiles. For the diode array, only two dose points are sampled in the high dose‐gradient region, which precludes any meaningful comparison. Shifting the detector with respect to the reference point did not result in an increased number of high dose gradient measurement points.

## 4 DISCUSSION

A CMOS array was characterized in terms of its angular dependency and a LUT was formed which corrected for the dependence. Next, the beam path files were used to calculate the beam angle with respect to the detector for each beam. Gamma comparison between TPS dose distributions and CMOS images was performed with and without the angular correction. Note that the dose linearity, dose‐rate dependence, and beam profile comparison against TPS has already been performed previously by Padelli et al.^8^ As mentioned above, Padelli et al. study did not account for the angular dependency and had to discard beams with angles >30°–40° while performing patient specific QA. The magnitude of the error can be large as seen in Figure 4, where a two‐fold increase was seen in the gamma passing rates. It should be noted that Padelli et al. also reported source to axis distance (SAD) or dose per pulse dependence. The SAD correction was not applied in this study, as the correction was assumed to be negligible.

The CMOS array was also compared with a diode array and gafchromic film. Excellent agreement can be seen with film, indicating that the array can provide film‐class spatial resolution, while also enabling real‐time analysis of data. For the diode array, only a few points are sampled for the 5 mm beam in the high dose region. Even though the software associated with the array reported a gamma passing rate of 98%, the analysis is not as meaningful as the array sampled only a few points. The data and angular correction methodology presented here validates the viability of using the CMOS array of Cyberknife patient QA. Comparable resolution to film, along with large detector array size make this device a valuable resource for clinics which routinely deliver 5 mm cones plans or large field MLC plans. The methodology presented for angular correction is general and can be applied to other detectors as well, which may have angular dependency.

## 5 CONCLUSION

A methodology for determining the Cyberknife beam angle was developed and validated. A high‐resolution CMOS array with angular correction was demonstrated to be superior to other available tools because of its sub‐millimeter spatial resolution and immediate read out of the results. The array will enable QA of plans with small fields.

## AUTHOR CONTRIBUTIONS

Jochen Krimmer, Laszlo Zalavri, Xuejun Gu, Lei Wang, and Cynthia Fu‐Yu Chuang contributed to the design and implementation of the research. Muhammad Ramish Ashraf acquired data and took the lead in writing the manuscript.

## CONFLICT OF INTEREST STATEMENT

The authors declare no conflicts of interest.

## Supporting information

Supporting Information
