# Supplementary material for: Angular correction methodology and characterization of a high‐resolution CMOS array for patient specific quality assurance on a robotic arm linac
Source: J Appl Clin Med Phys. 2023 Aug 2;24(11):e14110. doi: 10.1002/acm2.14110 (PMC10647992; doi:10.1002/acm2.14110)
Supplement: Supplementary file 1 — Supporting Information [file ACM2-24-e14110-s001.docx]

Supplementary Data

Table S1: Comparing individual beams for the 5 mm fixed cone plan with the reference dose obtained from the TPS.

| **Beam #** | **Angle °** | **TPS Dose (cGy)** | **Dose Uncorrected (cGy)** | **Dose Corrected (cGy)** | **Uncorrected Difference (%)** | **Corrected Difference (%)** |
| --- | --- | --- | --- | --- | --- | --- |
| **2** | 50.94 | 53 | 46.17 | 47.75 | -12.89 | 9.91 |
| **3** | 50.92 | 17 | 16.57 | 17.13 | -2.53 | -0.76 |
| **22** | 107.91 | 41 | 35.82 | 43.31 | -12.63 | -5.63 |
| **26** | 90.06 | 51 | 40.32 | 51.36 | -20.90 | -0.71 |
| **43** | 39.15 | 9 | 9.45 | 9.57 | 5.00 | 6.30 |
| **45** | 49.09 | 44 | 39.78 | 42.71 | -5.97 | -2.93 |
| **35** | 60.04 | 23 | 20.04 | 21.24 | -12.87 | 7.63 |
| **37** | 54.62 | 14 | 12.67 | 13.14 | -9.50 | 6.14 |
| **56** | 84.18 | 66 | 50.84 | 64.68 | -22.97 | -2.0 |
| **65** | 108.99 | 34 | 25.21 | 33.11 | -25.85 | 2.62 |
